# Genu Valgum

**Published:** 1909-04-24

**Authors:** 


### Surgery.

### GENU VALGUM.

Genu Valgum, or knock-knee, is a deformity of the lower extremity, in which the internal malleoli are separated by a variable distance when the legs are fully extended at the knees. It may arise from one of several causes, but the varieties most commonly seen are those which are due respectively (1) to rickets and (2) to the rapid growth associated with adolescence, accompanied, as it is, by corresponding weakness of the periarticular structures. More rarely cases are seen which can be definitely ascribed to injury, inflammation, or paralysis.

Rickets accounts for the great majority of cases, but the exact manner in which the deformity is caused differs according to whether it comes on in childhood or later. When rickets causes genu valgum in the early years of infantile life before the child has begun to walk obviously no mechanical factor resulting from the assumption of the erect attitude can be called into play. In these cases the deformity is the direct result of rickety changes in the bone—*i.e.* bending of the shaft and increase in the thickness of the inner half of the lower epiphysis. But when it comes on in adolescent life, this simple explanation is not sufficient. Some authorities are of opinion that all adolescent cases are due to "delayed rickets"; but whether this be the case or not, it is at any rate certain that there is a mechanical element in their causation.

In the erect position the weight of the body is transmitted to the ground from the pelvis through the outer condyle of the femur to the sole of the foot. The responsibility for the prolonged maintenance of the upright position falls mainly on three structures, the ligament of Bigelow, the internal lateral ligament of the knee, and the structures supporting the arch of the foot. The former of these is one of the strongest structures in the body, and is quite adequate to withstand any strain that may be thrown upon it; but the internal lateral ligament of the knee is, comparatively speaking, weak, and tends to elongate, and thus genu valgum begins. The internal condyle lengthens secondarily, and the deformity thus becomes permanent. Later the foot is everted, the weight of the body being transmitted through the inner half of the arch, which is ill-fitted to bear the strain, and flat foot results. Advanced degrees of genu valgum are nearly always complicated by flat foot.

The chief results of knock-knee are a waddling, awkward gait, owing (1) to the weakness of the ligaments round the knee, and (2) to the liability of the internal condyles to knock against one another in the act of progression (hence the name of the condition). To avoid this the patient, as he brings his legs forward in walking, throws them outwards in a curious and characteristic manner.

The patient himself complains of difficulty in getting about quickly, and of the fact that he gets tired very readily after walking or standing for any length of time. The diagnosis is usually obvious as soon as the patient is seen to walk; but one word of caution is necessary. The patient may refer all his symptoms to the feet, complaining only of pain in the sole owing to the secondary pes planus, and the primary cause of his condition may thus be overlooked.

On examining the patient it will be found that when the legs are extended and the internal condyles are brought into apposition, the malleoli are separated by a variable distance. Cases have been recorded in which the separation was more than 18 inches. If the legs are flexed the deformity immediately disappears. At first sight this would seem somewhat difficult to account for; but the explanation is that the enlargement of the internal condyle takes place in the vertical measurement only; the antero-posterior diameter is unaffected.

The treatment of genu valgum differs according to the age of the patient. In children who have just begun to walk, and in whom there is a slight degree of separation of the malleoli, spontaneous rectification of the deformity will nearly always occur if the child can be prevented from walking. This is no easy matter; but it can generally be managed by applying splints to the legs, the lower ends of which come well below the feet. Children have been known to attempt to walk even with this impediment; but if the projecting ends of the two splints are made of a different length, such a feat becomes well-nigh impossible. The only object of the splint is to keep the child off its legs. It is not necessary, nor is it desirable, to bandage the limb to the splint tightly in such a direction that a counteracting force is applied in opposition to the deformity. In children, in addition to the application of long splints, general treatment for the rickets must also be given, since genu valgum at this age is a manifestation of the disease in a more or less acute form. Such treatment resolves itself into the administration of cod-liver oil and directions for the proper feeding of the child.

If this treatment fails, osteotomy is the only proceeding likely to benefit the condition. In adolescent cases of any serious degree, it is generally advisable. But it should not be undertaken lightly. It should not be performed in a child under six years, nor unless there is a separation of the malleoli of at least three inches.

The operation itself is comparatively simple. The best operation is division of the shaft from the outer side. A sandbag is placed under the limb. A vertical incision is then made on the outer side. Its centre should correspond to a point two fingers' breadth above the upper border of the patella. The incision is carried down to the bone, and a keyhole saw introduced with which the bone is divided. Some surgeons prefer an osteotome, but a saw is less likely to slip and damage important structures. The wound is sewn up, and the operation is complete. It is better not to do both legs at the same time. In children it is preferable to put the limb up in a plaster or silicate bandage at the time of operation. In six weeks the plaster may be taken off and crutches commenced.

